# Inter-Strain Differences in Default Mode Network: A Resting State fMRI Study on Spontaneously Hypertensive Rat and Wistar Kyoto Rat

**DOI:** 10.1038/srep21697

**Published:** 2016-02-22

**Authors:** Sheng-Min Huang, Yi-Ling Wu, Shin-Lei Peng, Hsu-Hsia Peng, Teng-Yi Huang, Kung-Chu Ho, Fu-Nien Wang

**Affiliations:** 1 Department of Biomedical Engineering and Environmental Sciences, National Tsing Hua University, Hsinchu 300, Taiwan; 2 Department of Biomedical Imaging and Radiological Science, China Medical University, Taichung 404, Taiwan; 3 Department of Electrical Engineering, National Taiwan University of Science and Technology, Taipei 106, Taiwan; 4 Department of Nuclear Medicine, Chang Gung Memorial Hospital and University, Taoyuan 333, Taiwan; 5 Center for Advanced Molecular Imaging and Translation, Chang Gung Memorial Hospital and University, Taoyuan 333, Taiwan

## Abstract

Genetic divergences among mammalian strains are presented phenotypically in various aspects of physical appearance such as body shape and facial features. Yet how genetic diversity is expressed in brain function still remains unclear. Functional connectivity has been shown to be a valuable approach in characterizing the relationship between brain functions and behaviors. Alterations in the brain default mode network (DMN) have been found in human neuropsychological disorders. In this study we selected the spontaneously hypertensive rat (SHR) and the Wistar Kyoto rat (WKY), two inbred rat strains with close genetic origins, to investigate variations in the DMN. Our results showed that the major DMN differences are the activities in hippocampal area and caudate putamen region. This may be correlated to the hyperactive behavior of the SHR strain. Advanced animal model studies on variations in the DMN may have potential to shed new light on translational medicine, especially with regard to neuropsychological disorders.

The default mode network (DMN) of the human brain, which can be detected by monitoring the intrinsic low-frequency fluctuations in neural circuitry, has been widely investigated since its discovery more than a decade ago^1^. The DMN is postulated to support various self-referential functions like imagination^2^, conceptual processing^3^, and conscious awareness^4^. After the pioneering PET study by Raichle *et al.* it was further identified by Greicius *et al.*^5^ using fMRI, after which a number of other studies also confirmed its existence^2^,^4^,^6^,^7^,^8^,^9^. The DMN is regarded as a background functional organization of brain, one which processes the information received by the brain and is ready to responds to any environmental changes. Intriguingly, DMNs have also been shown to be present in non-human primates^10^. Moreover, Lu *et al.* showed that rat brains also have a DMN that is broadly similar to the DMNs of non-human primates and humans, a finding which suggests that DMNs appear to be a fundamental feature of the mammalian brain^11^. The demonstration of rat DMN thus offers a practical platform for exploring the role of the DMN in animal models. Recently, Shah *et al.*^12^ has demonstrated the strain-dependent differences in resting-state neuronal activity between mouse strains, including DMN-like network. In this study, we were interested in determining the degree of DMN variation among two specific rat strains with very similar genetic origins as our targets for fMRI scans.

The spontaneously hypertensive rat (SHR) was initially developed by Okamoto *et al.* as an animal model for studying hypertension^13^. It is an inbred strain that was established by selecting for hypertension in the Wistar Kyoto rat (WKY) strain. Thus, WKY rats are regarded as the most suitable control group for studying SHR rats. Although these two inbred strains have enormously similar genes, the expressions on cardiovascular and neuropsychological functions are quite different. Therefore, our question is: Is it possible to find the inherent variances in brain activities and organizations on these two rat strains? Investigations of these genetically similar strains could be somewhat analogous to the ongoing Human Connectome Project that focuses on twins and their non-twin siblings. Homozygous rats of an inbred strain are like twins, while the two strains with similar genetic origins, SHR and WKY, are comparable to non-twin siblings. Therefore, we set out to investigate the DMN of SHR and WKY rats. In this study, DMN and functional connectivity networks were analyzed through resting state functional MRI (rs-fMRI) scans using seed-based correlation analysis.

## Results

As noted, rs-fMRI was used to characterize the DMN of the rodent model in this study. In contrast with conventional fMRI methods, rs-fMRI is a task-free technique that monitors the intrinsic and fundamental activities of brain neural circuits^14^,^15^. We performed rs-fMRI scans using gradient echo echo-planar-imaging (EPI) sequence. For each subject, a total of 300 consecutive volumes were acquired with a temporal resolution of 1 second. An additional Data pre-processing was described in *Materials & Methods* part. To evaluate the DMN of rat, retrosplenial cortex (RSC) seed was selected and its time-varying signal curve was calculated. Then the correlation with the seed signal was carried out pixel-by-pixel to obtain a correlation coefficient map. The inter-subject variation is described in Supplementary Fig. S1. Among the nine subjects in each group, the nine correlation coefficient maps were subjected to one sample t-test against 0.3 and p < 0.0056 (Bonferroni-corrected) threshold was used to gated the final DMN map.

**Figure 1:**
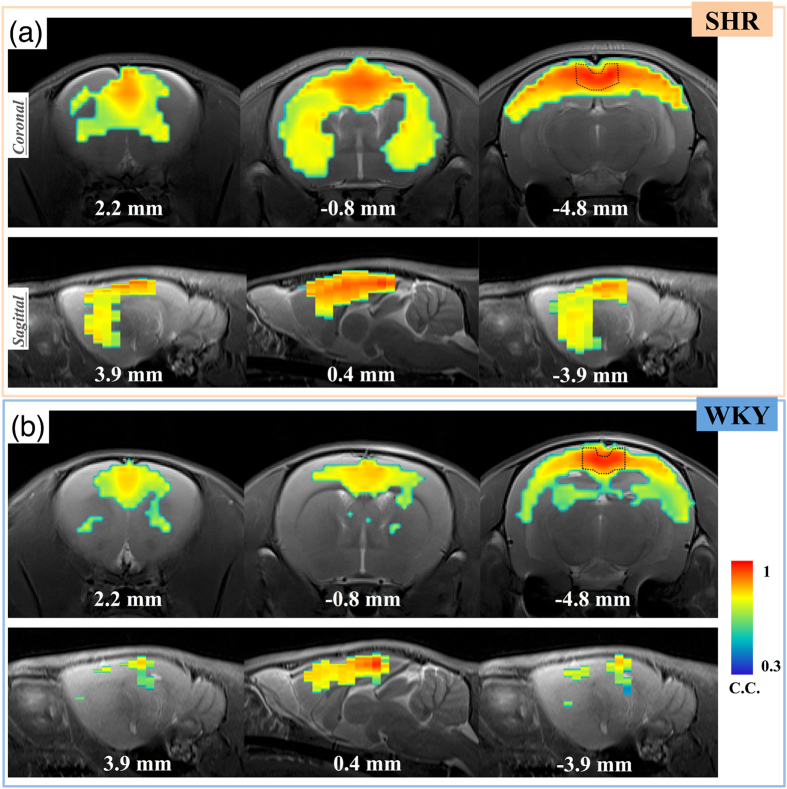
The DMN maps derived from RSC seeds. (Dash line ROI at Bregma −4.8 mm).The maps show the pixels with correlation coefficients significantly larger than 0.3 (p < 0.0056). The SHR rats presented clusters including the orbital cortex, cingulate cortex, posterior parietal cortex, auditory cortex, somatosensory cortex, and caudate putamen. On the other hand, the WKY rats showed a dissimilar pattern. The SHR rats showed widespread connection between the caudate putamen and RSC seed, while the WKY rats presented a pattern with hippocampal activity.

Figure 1 provides maps of the DMN based on RSC seed from 18 subjects (N = 9 in each group) overlaid onto T2 anatomic images. The SHR rats presented clusters including the orbital cortex, cingulate cortex, posterior parietal cortex, auditory cortex, somatosensory cortex, and caudate putamen, while the WKY rats showed a dissimilar pattern. Major differences were found with regard to two regions: the caudate putamen and the hippocampus area. The SHR rats showed a widespread connection between the caudate putamen and RSC seed, while the WKY rats presented a pattern with hippocampal activity. The correlation coefficients among the cingulate cortex area in the DMN map of SHR group were higher than those for the WKY group.

**Figure 2:**
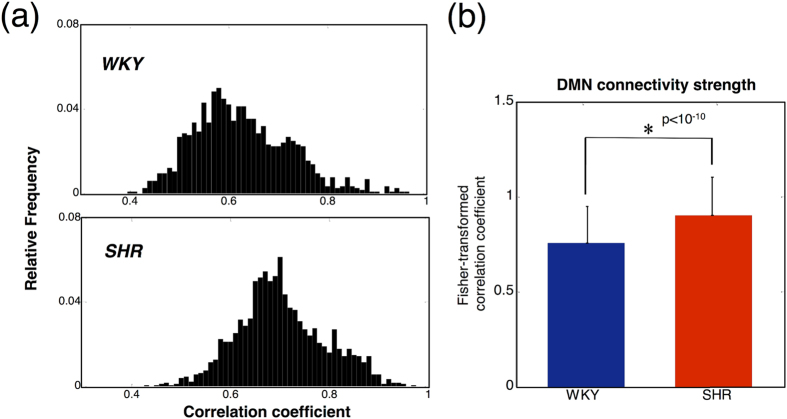
Comparison of the connective strength within the DMNs. (**a**) Histograms of correlation coefficients within the DMNs of the SHR and WKY rats. The differences in the distributions can be visualized. A quantitative comparison of the connective strength within the DMNs for the two strains is shown in **(b).** Correlation coefficients were transformed to Fisher’s z-scores. The SHR rats presented with significantly higher DMN strength (p < 10^−10^). The error bar denotes the standard deviation between voxels.

To further visualize the distribution of connectivity strength, correlation coefficient histograms of the respective DMNs for the two groups were generated (Fig. 2). The differences between the histograms of the SHR and WKY rats can be easily recognized. Fisher’s z transform was applied to quantify these differences, and the results showed that the SHR rats exhibited significantly higher connectivity strength (p < 10^−10^, two-sample t-test). This suggests that the DMN in the SHR rats is generally more active than the DMN in the WKY rats. The pixel counts of the network for the SHR and WKY rats were 1739 and 1160, respectively.

**Figure 3:**
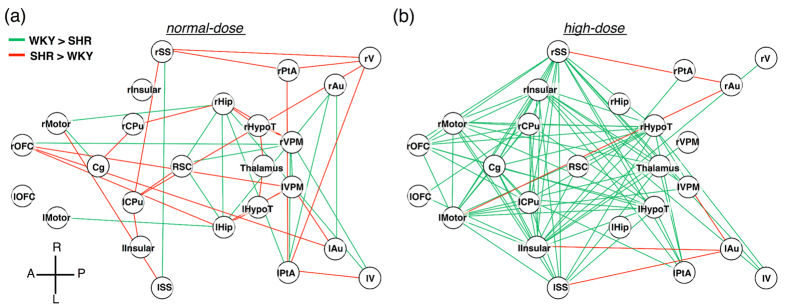
Network analysis based on 25 a priori ROIs. Lines were depicted in case the correlation coefficients between the two groups were significantly different (p < 0.0056). **(a)** Network analysis under normal-dose anesthesia. **(b)** Network analysis under high-dose anesthesia. The graphical visualizations demonstrate that the respective connective organizations in the SHR and WKY rats were unequal under both normal- and high-dose anesthesia.

A graphical visualization of a network analysis under normal-dose anesthesia based on 25 a priori ROIs is shown in Fig. 3(a). Lines were drawn if the connection strength was significantly different. Consistent with the findings for the DMN, the connectivity between the RSC and the hippocampus was stronger in the WKY group. The connection between the hippocampus and motor cortex was also stronger in the WKY group. On the other hand, the SHR group appeared to have enhanced connectivity in cortico-striato-thalamo-cortical (CSTC) loops, including the thalamic system, orbital frontal cortex, somatosensory cortex, and caudate putamen. The graphical visualization demonstrates the contrasts in the connective organizations of the SHR and WKY rats under normal-dose isoflurane anesthesia.

To further uncover the fundamental organizations of brain connectivity and the responses to anesthesia in these two rat strains, we conducted intensive anesthesia experiments by administering a higher concentration of isoflurane. Figure 3(b) depicts the results under this high-dose anesthesia. In contrast to the results for the normal dose, the WKY rats showed much stronger connectivity over most of the ROIs than did the SHR rats, suggesting a larger global reduction of brain connectivity in the SHR rats than in the WKY rats.

**Figure 4:**
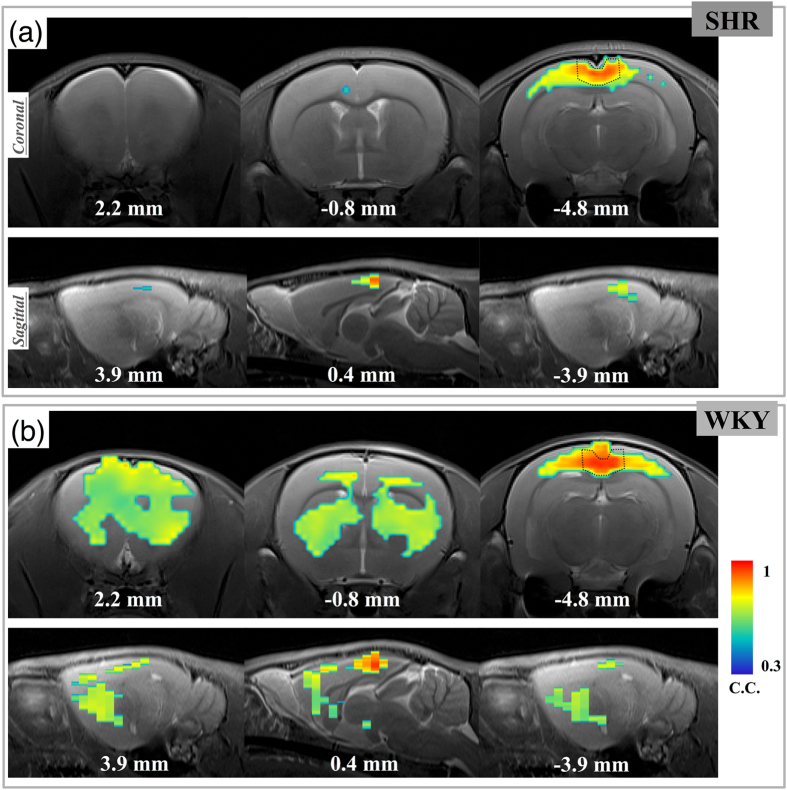
The DMN map under high-dose anesthesia derived from RSC seed. The map shows the pixels with correlation coefficients significantly larger than 0.3 (p < 0.0062). An obvious decrease of DMN was evident in the SHR group, while the DMN of the WKY group tended to be disrupted into a different organization. Note that activity in the caudate putamen region emerged in the WKY group.

To verify the response of brain activity to intensive anesthesia, the resting state networks (RSNs) based on RSC seed was evaluated and is shown in Fig. 4. The WKY rats exhibited activity in the caudate putamen region, while the activity patterns in the hippocampus and cingulate cortex were decreased in comparison to those found in normal-dose anesthesia group. The DMN pattern of WKY under intensive anesthesia tended to be disrupted into a sparse one. Among the SHR rats, meanwhile, activity was only found near the RSC region.

## Discussion

The DMN has been postulated to support various self-referential functions. Related to this, abnormality or dysfunction of the DMN has been considered relative to various psychophysiological diseases. The study of the DMN has thus become a potential means of disease investigation. In the present study, major differences between the SHR and WKY rats were found with regard to two regions: the caudate putamen region and the hippocampus area. Although the caudate putamen was not found to be part of the DMN of rats in a previous study^11^, we found a positive correlation between RSC seed and the caudate putamen in the SHR group. This correlation may have resulted from aspects such as animal strain, the ages of the animals used, and the inherent characteristics of the SHR rats. In the WKY group, the RSC derived DMN was found to cover the cingulate cortex, orbital cortex, posterior parietal cortex, auditory cortex, and even hippocampus, a finding which was consistent with the aforementioned previous study^11^. Since the WKY rats served as a control for the SHR rats in this study, we could infer that the presence of the caudate putamen area in the DMN of the SHR rats may reflect an inherent characteristic of the SHR rats.

In a previous report, the striatum region of SHR rats was shown to have impaired dopamine release^16^. The expression of the dopamine transporter (DAT1) gene was found to be disturbed in SHR rats, which would alter the uptake and reutilization of dopamine^17^. In our results, prominent striatal activity was observed in the DMN of the SHR group. It is anticipated that this dysfunction of the dopamine system in the striatal area may be related to the observed coherent activation of the caudate putamen in the DMN of SHR rats. In other words, the caudate putamen might participate in the DMN of SHR. In a study by Cao *et al.*^18^, a decrease in inverse connectivity between the putamen and DMN in attention deficit and hyperactivity disorder (ADHD) children was found. The enhancement of positive connectivity in our results and the decrease of inverse connectivity in Cao’s study appear to reflect comparable trends, which implies the potential of using SHR rats for future translational studies. With regard to anatomical evidence, a difference in the striatal volumes of SHR and WKY rats was also reported in a previous study^19^. The striatal volume in SHR rats was found to be smaller than that of WKY rats at 5 to 9 weeks of age. Though the reason for this variation remains unclear, the dissimilarity in striatal characteristics may be key information with regard to ADHD pathology. Since the putamen is mainly involved in the regulation of motor behavior and the caudate nucleus is related to social behavior, the symptoms of ADHD may be tied to this network abnormality. Many researchers have reported using SHR rats as an animal model of ADHD^16^,^17^. To the best of our knowledge, however, the present study is the first to show evidence of a different DMN between SHR and WKY rats.

The hippocampal area, which is in charge of learning, memory, spatial navigation, and emotional behavior, is an important brain structure for the consolidation of information. The RSC region is connected primarily to the rostrocaudal part of the hippocampus, and the caudoventral areas of the hippocampal formation are connected with the caudal parts of the RSC^11^. Neuronal projections of the hippocampal formation and the RSC suggest the involvement of memory and emotional behavior.

Our analysis of DMN connection strength also showed a distinct difference between the two groups. Specifically, the SHR rats exhibited higher connection strength within the DMN than the WKY rats did. Although the relatively high connectivity of the SHR group was partly due to the involvement of the caudate putamen region, the difference between the two groups was still significant even after excluding the contribution of the caudate putamen region. This suggests that the connection strength of the DMN in SHR rats is globally higher than that of the DMN in WKY rats. Increases in DMN strength have also been observed in chronic stress studies in both humans^20^ and rats^21^, as well as in depression and schizophrenia^22^. In schizophrenia, the hyperactivity of the DMN may be related to overly intensive self-referencing and impairments in attention and working memory. In SHR rats, meanwhile, the hyperactivated DMN may reflect the impairment of attention. Nevertheless, resting state studies on human ADHD have revealed a different result with regard to the DMN. Specifically, it has been shown that there is reduced DMN connectivity in cases of ADHD, including reduced connectivity in the medial prefrontal cortex and posterior cingulate cortex regions^23^,^24^. These studies suggest the possibility of a delay in neuromaturation in individuals with ADHD. Although our findings regarding the DMN of SHR rats were not entirely consistent with those found in human studies, we have demonstrated that DMNs in SHR and WKY rats are dissimilar, which suggests a new means of investigating functional differences in rodent brains. Our results may imply that, to a certain extent, the origins of ADHD symptoms in this rodent model may differ from the origins of ADHD in humans. These differences may be explained by the neuropathological basis of ADHD, which tends to have several possibilities and pathways^25^.

Consistent with the overall DMN findings, the connectivity between the RSC and the hippocampus was stronger in the WKY group (Fig. 3a). Interestingly, the connection between the hippocampus and the motor cortex was also stronger in the WKY group. As suggested in a previous study^26^, the hippocampus is tightly related to implicit motor sequence learning. Relatedly, recent human studies have demonstrated motor skill problems in children with ADHD^27^,^28^. Both of these findings imply that the decrease of hippocampal motor connectivity in the SHR group could play an important role in the ADHD behavior in SHR rats. On the other hand, SHR rats appear to exhibit enhanced connectivity in CSTC loops including the thalamic system, orbital frontal cortex, somatosensory cortex, and caudate putamen. The connectivity of CSTC loops has been reported to be either decreased or increased in several studies related to human ADHD^25^. Our findings may provide notable information regarding this animal model, and the underlying mechanisms may be clarified in future studies.

In order to simulate genuine “resting state” conditions, the introduction of anesthesia is often reduced as low as possible and muscle relaxants are usually given to prevent the motion of animal subjects. Resting state networks have been shown to be preserved under anesthesia^29^,^30^. A comparison of awake and anesthetized conditions has also been conducted by Liang *et al.*^29^. Although scanning conducted when animals are awake should be rationally near to “resting state,” the awareness of immobility would induce anxiety and stress which could affect the psychological state of animal subjects. This confounding factor could then influence the observed DMN. Furthermore, a recent study has demonstrated the impact of chronic stress on the RSNs of the rat brain^21^. The restraint-acclimating process conducted before rs-fMRI scanning makes it possible to temporarily alter the RSNs. Consequently, scanning of rats in anesthetized states was chosen in our study.

In this study, isoflurane/O_2_ mixture was used for animal anesthesia. Recent studies have shown variation in functional connectivity patterns under different kinds of commonly used anesthetics^31^,^32^. The use of pure oxygen would also lead to smaller BOLD changes than would be caused by the use of medical air^33^. The impact of different anesthetics on the DMN differences between SHR and WKY rats is an open question and may need further investigation. In Hutchison *et al.*’s work^34^, isoflurane dose-related alterations in the dynamic properties of functional connectivity in macaques were reported. The dynamic characteristics of RSNs under various anesthesia levels in SHR and WKY rats could be an interesting issue for future study.

Another issue is that we used 1.4% isoflurane for the normal anesthesia condition, which is higher than the 1% isoflurane used as a normal dose in other studies. In our experiments, however, we found that 1.4% isoflurane was the minimum dose required to maintain a stable respiratory rate in such young rats. We speculate that age may be a factor since Chemali *et al.*^35^ have reported that young adult rats have a higher dose requirement for isoflurane-induced loss of righting (LOR). Therefore, a 1.4% isoflurane/O_2_ mixture was used in this study to achieve a stable physiological condition during rs-fMRI scanning. The representative resting state BOLD signal-time curves are shown in Supplementary Fig. S2. An additional forepaw electro-stimulation experiment was also performed to verify the neuronal-BOLD responses under this anesthesia regime (see Supplementary Fig. S3). Furthermore, our findings on the DMN structure in the WKY group were similar to those reported in a previous study 11, which suggests the feasibility of our experimental procedure.

There were several considerations worth noting for the deep anesthesia group. It is generally understood that deep anesthesia could suppress neuronal responses and reduce the magnitude of hemodynamic responses (as shown in Supplementary Fig. S3). Besides, the baseline cerebral blood flows (CBF) of SHR and WKY rats were previously shown to be different under deep isoflurane anesthesia (2%) at around 12–16 weeks old^36^. Although SHR rats do not develop hypertension when still only 6 weeks old, the BOLD signal fluctuations seen in this study might have partially resulted from global vascular changes resulting from deep anesthesia, which could confound the evaluated RSNs. In spite of the aforementioned factors, however, we nonetheless sought to visualize the expression of brain connectivity under a relatively high dose of anesthesia. In other words, we did consider the issue of “resistance to anesthesia.” Interestingly, the patterns exhibited by the WKY and SHR rats were quite different. Under deep anesthesia, the pattern of the WKY rats covered the caudate putamen region, while the activity patterns in the hippocampus and cingulate cortex were decreased. As for the SHR rats, activity was only found near the RSC region. These changes might be due to the disruption of the original DMN, and due to the responses to anesthesia being different in these two strains. Moreover, the global vascular changes might also contribute to these results and may require further investigations. Disruptions of the DMN were found in previous studies regarding disorders of consciousness (DOC) such as comas and vegetative states^37^. Decreased connectivity was found in several brain regions, including the dorsal prefrontal cortex and anterior cingulate cortex^38^. Although the affected brain regions in this study were not similar to those found in DOCs, the utilization of high dose anesthesia provides a potential means of further investigating aspects of consciousness.

SHR rats have been shown to be a suitable model for ADHD^17^,^39^. SHR rats display behavioral characteristics such as hyperactivity, impulsivity, and poor performance in tasks requiring sustained attention, which are the major behavioral expressions of ADHD. Based on behavioral, genetic, and neurobiological criteria, Sagvolden *et al.*^39^ suggest that WKY rats serve as the best control compared with SHR rats. Since SHR rats present behavioral characteristics of ADHD, the expression of their spontaneous brain activity and neural organization could be a substantial issue in ADHD research. Abnormalities in RSNs in ADHD have been found in a variety of human studies^25^, mainly in terms of abnormality within the DMN^23^,^24^,^40^,^41^, abnormality in the interactions between the DMN and cognitive networks^24^,^42^,^43^,^44^, and abnormality in CSTC loops^18^,^45^.

SHR rats gradually present with stable and chronic hypertension at 2–4 months of age and show representative symptoms of human hypertension. Since hypertension is a confounding factor, we used animals at 6 weeks of age to exclude its influence. The hemodynamic parameters of SHR and WKY rats have been investigated in previous studies. While Leoni *et al.*^36^ found that SHR rats had a higher baseline CBF than control animals under 2% isoflurane, a more recent study indicated that there was no difference in the baseline CBF values between SHR rats and WKY rats under ~1% isoflurane^46^. Both of those studies used animals that were more than 12 weeks old, when the hypertensive state in SHR is already fully established, such that hypertension would influence the CBF autoregulation. Without the impact of hypertension in our study, however, we speculated that the CBF could be maintained within a certain autoregulatory range. Although the systolic blood pressure has previously been shown to be slightly higher in SHR rats than in WKY rats at 6 weeks old^47^, the CBF difference at 6 weeks old is likely to be limited. Also, an additional experiment showed that the stimulus-evoked BOLD signal changes were similar in the two strains (Supplementary Fig. S3). This suggests that the influence of non-neuronal factors on BOLD activities should have been minor, and so we speculated that the observed between-group differences in DMN were at least primarily due to differences in brain activity.

Several volumetric studies have found abnormalities in the brain regions of adult SHR rats as compared with WKY rats. Compared with WKY rats, SHR rats showed volume reductions in various regions, including the prefrontal cortex, occipital cortex, and hippocampus, and showed enlargement in ventricular volume^48^,^49^,^50^. Although these studies focused on adult rats and thus are not totally comparable with our study, the absence of hippocampal activity in the results for our SHR rats may be associated with the hippocampal volume reduction in adult SHR. As mentioned above, the striatal volume of 5-week old SHR rats was found to be significantly smaller than that of WKY rats in a recent study^19^. The striatal volume of 6 to 9 weeks of age SHR rats was also found to be smaller in that study. This difference may be related to the striatal activity presented in the DMN of SHR rats in our study. As a validated animal model of ADHD, SHR rats are suggested to present abnormalities in the regulation of dopaminergic function, including the impairment of dopamine release in the prefrontal cortex and caudate putamen^17^,^51^. Such dopaminergic dysfunction will result in abnormal signal transmission between neurons, which may alter the functional networks of the brain. Although the usage of SHR and WKY rats for ADHD research has been debated^17^,^51^ due to the hypoactivity of WKY rats, in this study we mainly focused on the network differences between these two strains.

Another issue is the comparison of functional networks with commonly used rats, including the Sprague-Dawley (SD) rat and the Lewis rat. Although SHR and WKY rats are usually used and tested together, functional network variability among other strains can be further investigated in the future. The strain-related differences in mouse brain network were recently described in Shah *et al.*’s paper^12^, including striatum region and DMN-like network. Based on our result, the inter-strain difference should be found in the commonly used rat strains.

Since it has been suggested that the striatal volumes of SHR and WKY rats would be different, it is possible that the results of the ROI connectivity analysis may have been partially affected accordingly. Moreover, the imaging spatial resolution in this study was 469 × 469 μm^2^ with 1-mm slice thickness, which limited the ability to differentiate adjacent brain structures. The spatial smoothing process with a 1-mm full width at half maximum Gaussian kernel would also limit the accuracy in smaller brain regions. The partial volume effect from neighboring regions like the auditory cortex would thus affect the observed activity of the hippocampus.

In spite of the considerations mentioned above, this work has demonstrated the dissimilarity between the DMNs of SHR and WKY rats. These differences suggest the possibility of further neuropsychological research on rodent models through rs-fMRI techniques.

## Materials and Methods

### Animals

All the animal experiments were approved by the National Tsing Hua University Institutional Animal Care and Use Committee and were carried out in accordance with the approved guidelines. Six-week-old SHR rats (n = 9, male, 141–170 g) and WKY rats (n = 9, male, 151–189 g) were used in this study. Before the onset of hypertension at 10–12 weeks of age, SHR rats have previously been shown to exhibit hyperactivity at 3–4 weeks of age^17^. Consequently, 6-week-old rats were chosen in this study to exclude the confounding factor of hypertension.

### Magnetic resonance imaging

All animals were scanned using 7-Tesla Bruker Clinscan with a volume coil for signal excitation and a brain surface coil for signal receiving. Anesthesia was induced with around 1.4–1.5% isoflurane mixed with O_2_ at flow rate of 1 L/minute. For each rat, the monitored respiratory rate was 65–75 breaths/min throughout the whole scanning period, and body temperature was maintained at 37 °C by a temperature-controlled water circulation machine. For the rs-fMRI experiments, 300 consecutive volumes with 11 coronal slices were acquired using gradient echo echo-planar-imaging (EPI) with TE/TR = 20 ms/1000 ms, FOV = 30 × 30 mm^2^, matrix size = 64 × 64, and slice thickness = 1 mm. Anatomical images were obtained by turbo-spin-echo (TSE) with scanning parameters of TE/TR = 14/4000, FOV = 30 × 30 mm^2^, matrix size = 256 × 256, slice thickness = 1 mm, number of average = 2. To examine the effect of deep anesthesia, 2.5~2.7% isoflurane mixed with O_2_ was introduced, and the monitored respiratory rate was 40–45 breaths/min throughout the whole scanning period.

### Data processing

The image registration process was carried out using the Automated Image Registration package (AIR3.0, http://bishopw.loni.ucla.edu/air5/)^52^,^53^ by 2-D affine 6-parameter model. Slice timing and spatial smoothing (Gaussian kernel, full width at half maximum = 1.0 mm) was performed on SPM8 software (http://www.fil.ion.ucl.ac.uk/spm/software/spm8/). Temporal detrend and bandpass frequency filtering (0.002–0.1 Hz) were conducted through the REST toolkit (REST v1.6, http://www.restfmri.net/forum/REST_V1.6)^54^. Seed-based and other statistical analysis was performed by self-designed Matlab (The Mathworks Inc., MA, USA) scripts. To determine the DMN of a rat, the retrosplenial cortex region at Bregma −4.8 mm was chosen as the seed based on Paxinos coordinates^55^. The RSC region is one of the major hubs of the DMN in rodents^11^, which corresponds to the posterior cingulate cortex in primates. Based on the averaged signal time course of for the RSC region, Pearson’s correlation coefficients were calculated pixel-wise across all the slices to generate a connectivity map. The correlation coefficients were transformed to z scores by using Fisher’s z-transformation and averaged across the nine subjects. Then the averaged z-score map was then transformed back to a correlation coefficient map. Among the nine subjects in each group, the nine correlation coefficient maps were subjected to one sample t-test against 0.3 and p < 0.0056 (Bonferroni-corrected) was used to threshold the final correlation coefficient map (the final DMN map). Histograms of the correlation coefficient map were then made to compare the connectivity distributions. To assess the difference between the DMN connectivity strength, the correlation coefficients from the final DMN map were transformed to z-scores and subjected to two-sample t-test for comparison, which is similar to Liang *et al.*’s study^29^. A higher z-score value stands for higher connectivity strength. To further assess the connection networks, 25 a priori ROIs were defined as seeds according to Paxinos coordinates^55^ for comparison. A 25 ×25 matrix of correlation coefficients was calculated for each rat. Connection strength differences were determined by comparing the correlation coefficients between the SHR and WKY rats using two-sample t-test with a threshold of p < 0.0056 (Bonferroni-corrected). For the high-dose anesthesia group, one of the subjects was removed from the analysis due to the presence of motion. Hence a total number of eight subjects in each group were chosen for pre-processing and analysis. The processing procedures were identical to those use for normal-dose anesthesia except for the significance level, which was set at p < 0.063 for these 8-subject groups.

## Additional Information

**How to cite this article**: Huang, S.-M. *et al.* Inter-Strain Differences in Default Mode Network: A Resting State fMRI Study on Spontaneously Hypertensive Rat and Wistar Kyoto Rat. *Sci. Rep.* **6**, 21697; doi: 10.1038/srep21697 (2016).

## Supplementary Material

Supplementary Information
