# Supplementary material for: Inter-Strain Differences in Default Mode Network: A Resting State fMRI Study on Spontaneously Hypertensive Rat and Wistar Kyoto Rat
Source: Sci Rep. 2016 Feb 22;6:21697. doi: 10.1038/srep21697 (PMC4761976; doi:10.1038/srep21697)
Supplement: Supplementary Information [file srep21697-s1.pdf]

Supplementary Information

Inter-Strain Differences in Default Mode Network: A Resting State fMRI Study on Spontaneously Hypertensive Rat and Wistar Kyoto Rat

Sheng-Min Huang[1], Yi-Ling Wu[1], Shin-Lei Peng[2], Hsu-Hsia Peng[1], Teng-Yi Huang[3], Kung-Chu Ho[4] and Fu-Nien Wang[1*]

[1] Department of Biomedical Engineering and Environmental Sciences, National Tsing Hua University, Hsinchu 300, Taiwan

[2] Department of Biomedical Imaging and Radiological Science, China Medical University, Taichung 404, Taiwan

[3] Department of Electrical Engineering, National Taiwan University of Science and Technology, Taipei 106, Taiwan

[4] Division of Nuclear Medicine, Chang Gung Memorial Hospital, Taoyuan 333, Taiwan

[*]Correspondence to Fu-Nien Wang: fnwang@mx.nthu.edu.tw

Supplementary Figure S1. The map of coefficient of variation for final DMN.

Supplementary Figure S2. The representative resting state BOLD signal-time curves.

Supplementary Figure S3. The forepaw electro-stimulus evoked BOLD signal changes in S1FL under 1.4% and 2.5% isoflurane anesthesia.

Figure S1

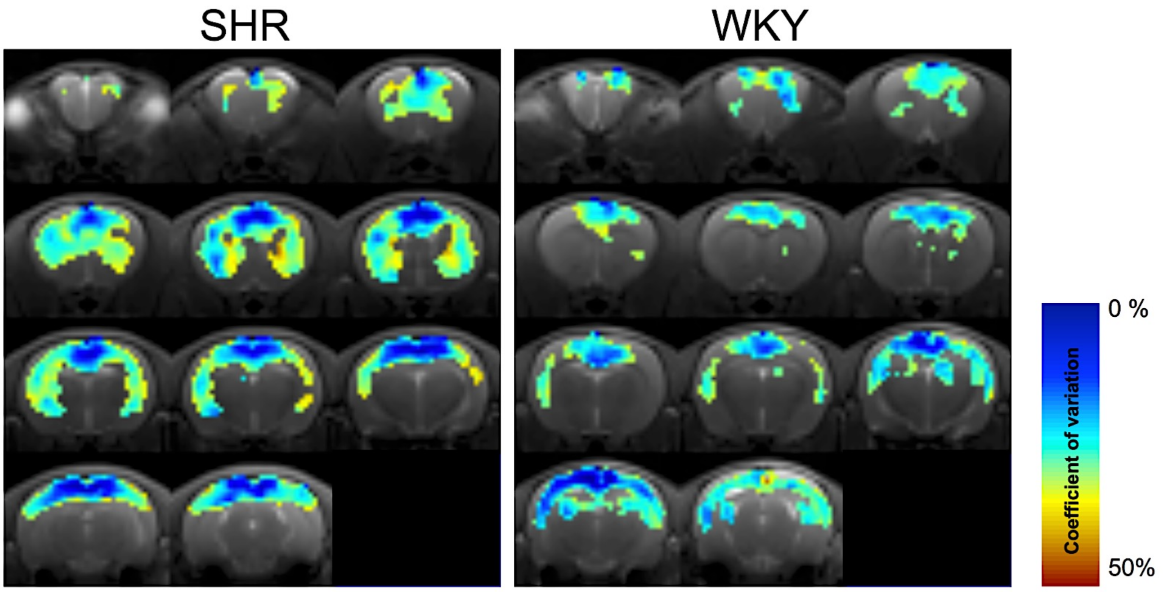

Figure S1: The map of coefficient of variation for final DMN. Color image values are percentage variation around the mean correlation coefficient in the nine subjects under normal anesthesia. Blue indicates small variation around the mean; red indicates larger variation.

Figure S2

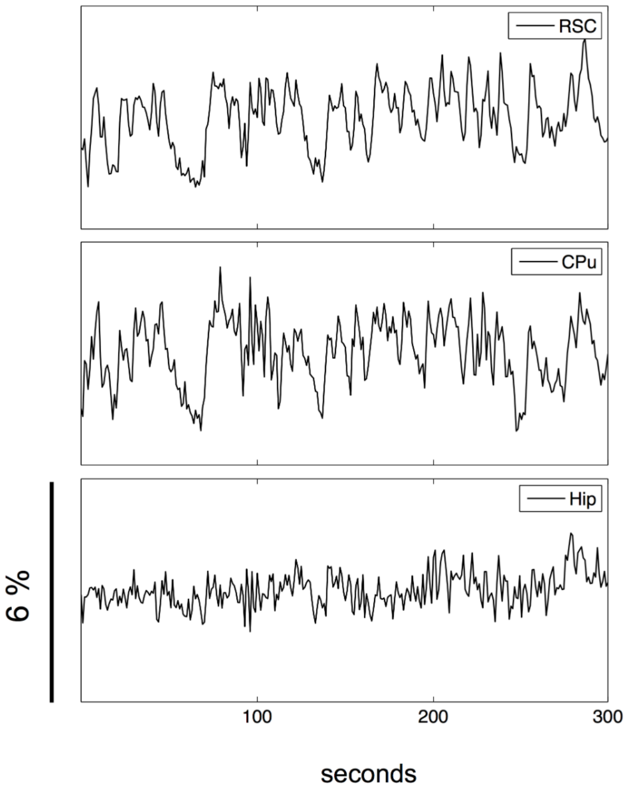

Figure S2: The representative resting state BOLD signal-time curves. The signal curves of three brain regions, the RSC, CPu, and hippocampus, of an SHR rat are shown. It is clear that the signal curves of the RSC and CPu are similar, while the hippocampus presents a different pattern. The ROI selection was based on the Paxinos atlas.

Figure S3

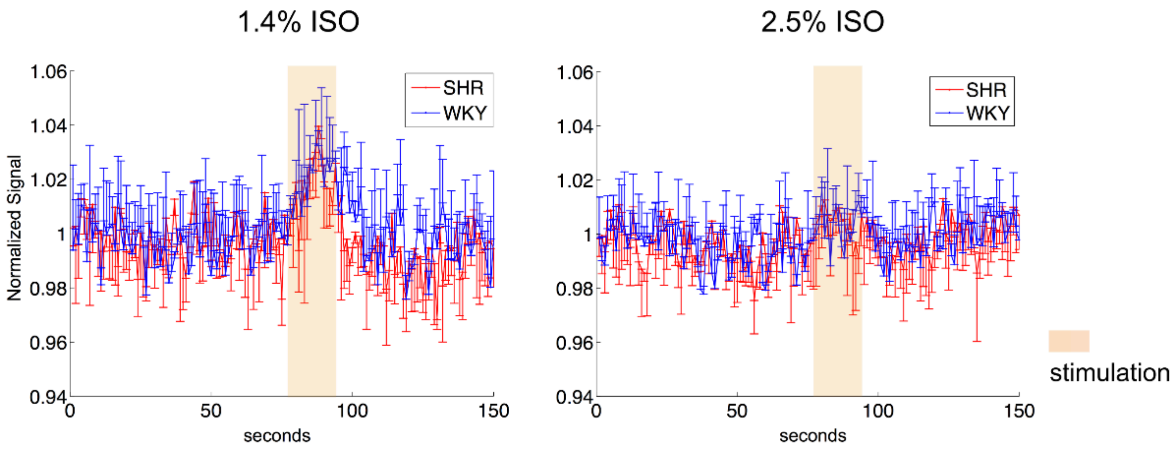

Figure S3: The forepaw electro-stimulus evoked BOLD signal changes in S1FL under 1.4% and 2.5% isoflurane anesthesia. The stimulus evoked signal change under 1.4% isoflurane was around 3%, which could confirm the neuronal-BOLD response. As to high dose isoflurane anesthesia, the BOLD signal change was not obvious. Stimulation parameters were 0.33ms pulse width, 3mA current at 3Hz frequency, 75s off and 15s stimulation on left forepaw. The errorbars were the standard deviation among three subjects.
